# An Innovative Food Processing Technology: Microwave Electrodeless Ultraviolet, Luminescence Mechanism, Microbial Inactivation, and Food Application

**DOI:** 10.3390/foods13244110

**Published:** 2024-12-19

**Authors:** Shuqi Chang, Zhaoyi Zhang, Qin Liu, Haixia Wu, Alideertu Dong

**Affiliations:** 1 College of Chemistry and Chemical Engineering, Inner Mongolia University, Hohhot 010021, China; 13173352065@163.com (S.C.); 15044819325@163.com (Z.Z.); 2 Engineering Research Center of Dairy Quality and Safety Control Technology, Ministry of Education, Inner Mongolia University, Hohhot 010021, China; 3 Inner Mongolia Tailida Dairy Co., Ltd., Hohhot 010010, China; lqsnt@163.com; 4 National Center of Technology Innovation for Dairy, Hohhot 010110, China

**Keywords:** microwave electrodeless ultraviolet (MWUV) sterilization technology, microwave discharge electrodeless lamps (MDEL), sterilization mechanism, food sterilization, food digestion

## Abstract

Microwave electrodeless ultraviolet (MWUV) technology, as an emerging food processing technique, has garnered growing attention in the realm of food science in recent years. Based on different application requirements, MWUV equipment types are categorized as microwave oven reactor, continuous-flow UV-microwave reactor, coaxially driven MWUV reactor, and complete ultraviolet reactor. The luminescence properties of MWUV equipment depend on their filler gas; mercury is commonly used as a filler gas to produce a wavelength at 253.7 nm for food non-thermal sterilization. The microbial sterilization effect of MWUV is primarily attributed to the synergistic action of microwave and ultraviolet (UV): MWUV enhances reactive oxygen species (ROS) production, disrupts the cell membrane structures of bacteria, leads to bacterial endosome leakage, and induces nucleic acid damage. MWUV extends food shelf-life by eliminating microorganisms without significantly altering food quality compared with traditional thermal sterilization methods. Additionally, MWUV, combined with digestion reagents such as HNO_3_ and H_2_O_2_, can effectively enhance the digestion of food samples to detect essential and toxic elements. Studies on MWUV technology hold broad potential in the food industry, with promising implications for food safety and consumer demand for high-quality food. Future research may focus on optimizing the equipment parameters and integrating with other food processing technologies to facilitate further development and application of MWUV.

## 1. Introduction

The processing and preservation of food have consistently posed a significant challenge for the food industry in managing microorganisms and extending food shelf life while minimizing quality degradation [1,2,3]. Food sterilization treatment aims to eliminate microorganisms and ensure food safety, and includes thermal, photochemical, and sonodynamic therapy (SDT). Nevertheless, traditional sterilization techniques, like thermal sterilization, have the potential to greatly influence the nutritional and sensory characteristics of food products [4,5]. In recent years, non-thermal sterilization techniques have garnered increased attention due to their reduced impact on food nutrients [6], such as high-pressure processing (HPP) [7], pulsed light (PL) [8], ultraviolet (UV) [9], low temperature plasma (LTP) [10], and pulsed electric fields (PEF) [11].

Microwave (MW) sterilization extends food shelf life by inactivating microorganisms through rapid heating with MW energy [12,13]. This process relies on the electromagnetic radiation features of MW, and the operated frequency ranges from 300 MHz to 300 GHz. When MW penetrates food, water molecules and polar substances (e.g., ions and polar molecules) undergo intense rotation and oscillation, generating thermal energy through ion migration and dipole rotation, which results in heat production [14,15]. This internal heating mechanism quickly elevates the food’s temperature to a level that effectively kills microorganisms. Compared with traditional thermal sterilization methods, MW sterilization is distinguished by rapid and uniform heating, which facilitates the inactivation of pathogens while maximizing the preservation of the food’s nutritional and sensory attributes [16,17,18]. Additionally, MW directly targets the food’s interior, rapidly bringing it to sterilization temperatures and effectively reducing the presence of bacteria, mold, and other pathogens.

Ultraviolet (UV) sterilization primarily operates through short-wave UV light, specifically UV-C (200~275 nm) [19,20]. UV sterilization functions by disrupting microbial DNA or RNA, inhibiting cell division and reproduction, and ultimately leading to microbial death [21,22]. UV-C is highly efficient with broad-spectrum sterilizing properties, requiring no chemical agents and producing no secondary pollution, making it widely applicable in food sterilization and preservation [9,23,24].

The initial applications of combined MW and UV technologies focused on pollutant degradation in wastewater and organic synthesis [25]. With further investigation into these combined technologies, their synergistic effects on microbial degradation were identified [26], revealing that the bactericidal action of MW and UV together enhances the biological effects seen with each technology alone. Nowadays, the synergistic usage of MW and UV in food processing has been established [27]. Research has demonstrated that a bulb or a lamp containing vapor and inert gasses can emit UV light within a MW field (e.g., in a MW oven), marking the development of the original MW electrodeless ultraviolet technology (MWUV)] [26]. Over time, MWUV devices have evolved to include combinations of MW and UV, as well as designs that shield MW, allowing only UV to be emitted [28]. As MWUV is a new type of non-contact sterilization technology, this article reviews the developments in this field, including MWUV equipment design, the effects of MWUV on food material processing and final product quality, and the degradation of microorganisms. This review aims to provide insights for future researchers interested in developing novel food processing methods with MWUV technology.

## 2. Microwave-Assisted Ultraviolet Equipment

### 2.1. Microwave-UV Reactor

**Figure 1:**
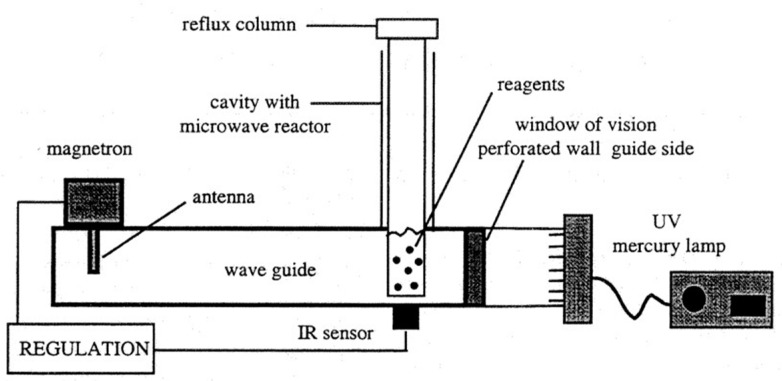
Scheme of the microwave-UV reactor [25].

The early combined applications of MW and UV are in the form of separate or combined use of two technologies. As shown in Figure 1, this microwave-UV reactor combines MW and UV technologies for organic synthesis and degradation reactions [25]. The operating principle relies on the selective heating of MW and the electronic excitation effect of UV light to provide a highly reactive environment and accelerate chemical reactions. The equipment is shielded by a specially designed microwave shielding window, which allows the equipment to independently evaluate the effects of MW and UV light while the reaction is being carried out. The rearrangement of o-benzoyloxyacetophenone and the degradation of humic acid in aqueous solution was experimentally confirmed to be enhanced via the combination of MW and UV.

### 2.2. Microwave Electrodeless Ultraviolet (MWUV) Equipment

Early studies on MUVW were mainly applied to wastewater treatment and the degradation of organic matter [29,30]. Several reports indicate that MW combined with UV can boost the efficiency of microwave photocatalysis. The luminescence of the microwave discharge electrodeless lamp (MDEL) is primarily dependent on the following sequence of events [31,32,33]:

- Microwave energy excitation: MDEL is powered by an external microwave rather than conventional electrodes. The microwaves (typically in the 2.45 GHz frequency range) enter the bulb’s interior through the bulb housing’s transparent material. The interior of the bulb is filled with a specific gas (e.g., mercury, xenon, or argon) and a small amount of inert gas. These gasses are excited by the microwave energy and ionized to form a plasma (Equation (1));
- Plasma generation: When microwave radiation penetrates a light bulb, gas molecules or atoms are excited and ionized under the action of the microwave field, producing high-energy plasma. The electrons in the plasma undergo collisions frequently with the gas molecules or atoms and transfer energy to the gas atoms, bringing them to an excited state (Equations (2) and (3));
- UV light emitting: Photons are released when the gas atom returns from its excited state to the ground state. The properties of these photons can vary depending on the gas medium in which they propagate. In MDEL, mercury is commonly used to emit UV light. This is because it emits UV light at around a wavelength of 254 nm, which is potent for sterilization and disinfection (Equations (4) and (5)) [28].

e^−^ + MW → e^−^*(accelerated), (1)

e^−^* + Ar → Ar^+^ + 2e^−^, (2)

e^−^* + Ar^+^ → Ar*, (3)

Ar* + Hg → Hg* + Ar,(4)

Hg* → Hg + hv(5)

#### 2.2.1. Microwave Oven Reactor

**Figure 2:**
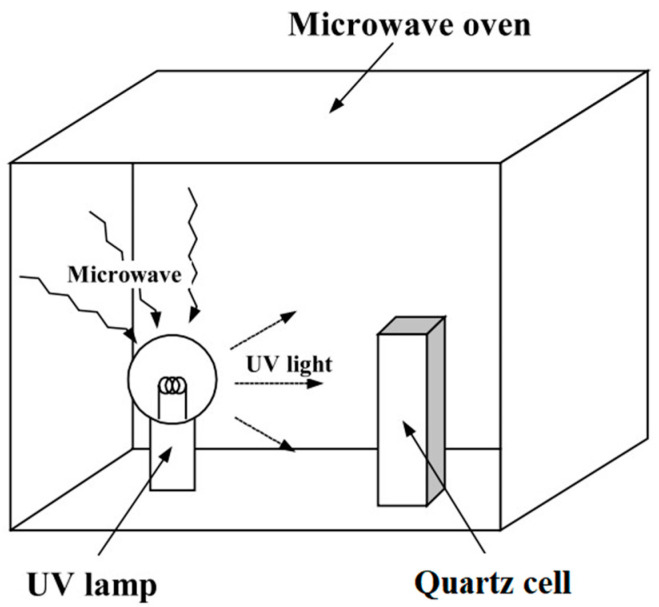
Microwave oven reactor [26].

Early MWUV reactors were mainly microwave oven reactors [34,35]. The reaction device is a simple combination of a microwave and an induction UV lamp placed inside a microwave oven, as shown in Figure 2 [26]. The device usually consists of a microwave source (e.g., a domestic microwave oven), an induction UV lamp, and a reaction chamber. The microwave oven provides microwave energy to excite an inert gas such as mercury or argon in the induction UV lamp to produce high-energy UV light for photocatalytic or disinfection reactions [36].

The design of the microwave oven is simple and easy to operate, rendering it suitable for utilization across a diverse array of research environments, facilitating the application of flexible UV irradiation [37]. However, the microwave oven’s size limits the reaction chamber’s volume. Therefore, the microwave oven is only suitable for smaller volumes of reactants or samples, whereas it is not sufficient for large industrial applications. At the same time, due to the relatively simple design of the equipment, part of the microwave energy may be absorbed or scattered by the reaction chamber wall or other components, resulting in the use of energy efficiency not reaching the theoretical maximum. Additionally, since a microwave oven reactor usually lacks a complex control system, it is difficult to accurately control the microwave intensity, ultraviolet intensity, and other parameters of the reaction environment, limiting its use in some acceptable chemical applications. Thus, further upgrading of microwave-driven UV equipment is still needed.

#### 2.2.2. Continuous-Flow UV-Microwave Reactor

**Figure 3:**
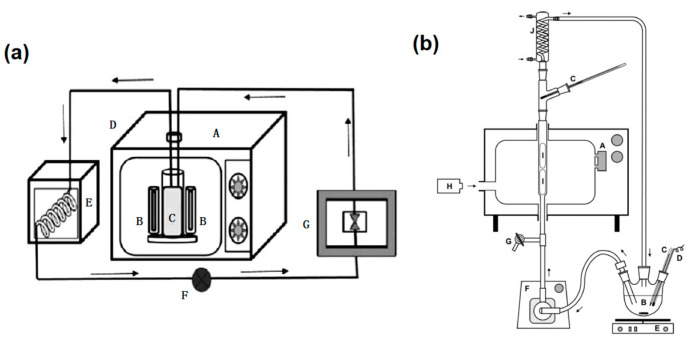
(**a**) Continuous-flow milk sterilization reactor: (A) microwave oven; (B) electrodeless UV lamp; (C) reactor; (D) Teflon pipes; (E) cooling chamber; (F) sample collector; (G) peristaltic pump [27]. (**b**) Continuous-flow photocatalytic degradation reactor: (A) modified microwave oven with magnetron; (B) glass reservoir with magnetic stir bar; (C) thermometer; (D) chloride ion-selective electrode and/or tube for air bubbling; (E) magnetic stirrer; (F) PTFE diaphragm pump; (G) outlet; (H) spectrometer with a fiber-optic probe; (I) glass tube with titania-coated EDLs; (J) cooling condenser. (Arrows indicate the flow direction) [38].

The continuous-flow UV-microwave reactor is designed as a circulating flow reactor, which allows the reactants to flow continuously through the microwave heating and UV radiation zones to achieve uniform treatment while avoiding overheating problems and enhancing the efficiency and stability of the reactor. The reactor is typically used to treat liquids [38,39]. As shown in Figure 3a, the microwave oven was a modified domestic model operating at a frequency of 2450 MHz and a power output of 1200 W. Two holes, each with a diameter of 5 mm, were drilled into the ceiling of the oven to accommodate Teflon pipes for inlet and outlet purposes. These pipes were connected to a peristaltic pump, and the pump speed was adjusted to allow a continuous flow of samples into the sample holder to sterilize milk [27]. As shown in Figure 3b, a glass tube with MEDL coated in thin layers of nanoporous titanium (IV) oxide was used to set up a continuous-flow UV-microwave reactor. It examined how operational parameters affected the TiO_2_/UV/MW process’s ability to photocatalytically degrade aqueous mono-chloroacetic acid (MCAA).

#### 2.2.3. Coaxially Driven MWUV Reactor

**Figure 4:**
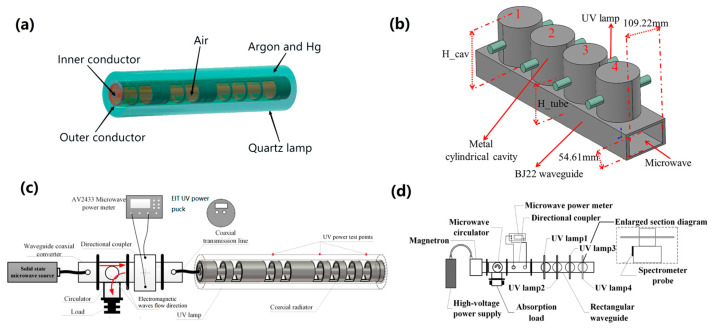
(**a**) Diagram of the cylindrical slot waveguide structure [31]; (**b**) diagram of the rectangular slot waveguide structure [41]; (**c**) diagram of the cylindrical slot waveguide reactor [31]; (**d**) diagram of the rectangular slot waveguide reactor [41].

A thin slot is added to the waveguide’s sidewall to create the waveguide slot radiator. It has strong construction, high power-bearing capacity, low loss, and steady performance [31]. The coaxially driven MWUV reactor, developed based on the mentioned slots, has two main structures. One is a cylindrical slot waveguide structure [31,40], as shown in Figure 4a. Several slots around the cylindrical slot waveguide structure are jacketed with a quartz tube. The quartz tube, usually filled with argon and Hg, is shielded from electromagnetic waves via a metal mesh. To excite the plasma lamp, the slots in the coaxial waveguide facilitate the coupling of electromagnetic waves, and the corresponding reactor is shown in Figure 4c. The other one is the rectangular slot waveguide structure [41], as shown in Figure 4b. Several slots are cut in the metal rectangular waveguide, and a metal cavity with a UV lamp is added to each slot. The slots of the rectangular waveguide enable the electromagnetic waves to couple into the metal cylindrical cavity, exciting the plasma lamp. Figure 4d shows the overall diagram of the experimental system.

The coaxially driven MWUV reactor is developed to tackle the issue of device size by achieving miniaturization without compromising on power [38]. By incorporating a coaxial slot structure, the device is able to reduce its footprint while maintaining the power output of the UV lamp. This innovative design approach results in a more compact device without sacrificing performance.

#### 2.2.4. Complete Ultraviolet Reactor

A complete ultraviolet reactor is a reactor in which microwaves excite the UV but shield the microwaves using unique materials [42]. The microwave induction UV reactor achieves the shielding of microwaves by the following methods:

- Metal shielding: Metals have good reflective properties for microwaves and can effectively block microwave leakage. In reactor design, metal shielding can surround the entire microwave source and reaction area, thus preventing microwaves from escaping and ensuring safety [43];
- Waveguide structure optimization: Through the specially designed microwave waveguide structure, the microwave energy can be concentrated in a specific reactor area so that the microwaves only act inside the reaction chamber without leaking to the outside world. This not only improves the reaction efficiency but also reduces the microwave interference from the external environment [44,45];
- Wave-absorbing material: In some designs, reactors also use wave-absorbing materials. These materials absorb excess microwave energy and convert it to heat, reducing microwave leakage [37];
- Faraday cage effect: Some microwave reactors are designed as Faraday cage structures, which utilize conductive materials to form a closed electromagnetic shield to block the escape of microwaves. This type of shielding is typically used in high-power microwave systems to ensure that the microwaves do not affect the surrounding environment [46].

Through these technical means, the microwave induction UV reactor can effectively shield microwaves without affecting the propagation of UV radiation, ensuring the safety and operational efficiency of the equipment. This type of reactor is also a new type of non-thermal sterilization equipment due to the shielding of microwaves.

Table 1 presents a summary of four types of MWUV equipment.

### 2.3. Microwave Discharge Electrodeless Lamp (MDEL)

**Figure 5:**
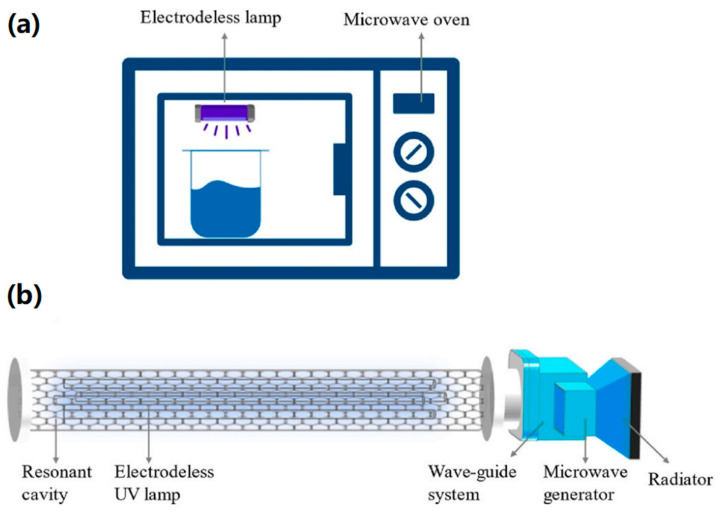
Two types of MDEL. (**a**) A microwave oven structure; (**b**) a resonant cavity structure [28].

There are two main types of MDEL: microwave-oven-type electrodeless lamp (MOTEL) and resonant cavity electrodeless lamp (RCEL) [28]. As shown in Figure 5a, MOTEL mainly puts the electrodeless ultraviolet lamp in the microwave oven; the gas inside the UV lamp receives microwave excitation, which will produce UV light. This type of UV lamp mainly uses microwave and UV synergistic effects [47]. MOTEL is simple and easy to operate, but it is not conducive to separating UV and microwave, and so the two physical fields often coexist. As shown in Figure 5b, the structure of the RCEL is a unique microwave generator that continuously and steadily transmits microwaves to a resonant cavity through a microwave waveguide system. The cavity is a well-designed mesh structure, which confines the microwaves inside and facilitates the reaction with the electrodeless lamp to produce UV [31]. This type of UV lamp can realize the separation of microwave and UV, and at the same time, it is convenient to regulate the process parameters of the equipment.

The filling substance is the determining factor in the radiant properties of a MDEL. The specific advantages of MDEL are widely accepted, including ease of manufacture with a competitive price point, an extended operational lifespan, and exceptional light efficiency. Furthermore, the flexibility of shape design inherent to MDEL renders it more advantageous for industrialized applications. As a consequence, it has been developed to replace traditional UV mercury lamps [48]. The filler gas in the MDEL determines its luminescence characteristics. Table 2 shows the filler gas and the emission wavelength of MDEL.

## 3. Sterilization by MWUV

### 3.1. Bactericidal Mechanism

The degradation of microorganisms by MWUV primarily utilizes the synergistic bactericidal effects of UV light and microwaves. The action of microwaves is mainly through their thermal effect, which kills microorganisms via dielectric heating. This process occurs when microwaves interact with polar molecules within the substance, like H_2_O. The rapid rotation and resultant friction of these molecules generate heat. When applied to the surface of food or other objects, this heat disrupts microbial proteins and cell membranes and induces cell dehydration, thereby achieving sterilization [54,55,56]. The non-thermal effects of microwaves have garnered significant debate in recent years [57,58]. Studies have demonstrated that microwave treatment can be more effective in killing bacteria than conventional water bath sterilization at equivalent or lower temperatures, possibly due to the non-thermal effects on microbial cell membranes, proteins, and DNA. However, the mechanisms underlying these non-thermal effects are not yet fully understood, and their characterization remains incomplete. Nonetheless, the dual presence of thermal and non-thermal microwave effects, in conjunction with UV irradiation, is recognized as contributing to microbial degradation [57].

The synergistic sterilization effect of combining microwave and UV irradiation is evident from the enhanced microbial inactivation achieved when both methods are applied concurrently, compared to each method used individually. This synergy is particularly effective against microbial defense mechanisms, resulting in higher sterilization efficacy. Reactive oxygen species (ROS) are predominantly generated by the UV-induced irradiation of airborne oxygen and water [59]. These ROS, such as ·OH and ·O_2_^−^, induce oxidative stress, damaging intracellular biomolecules, including lipids, proteins, and DNA, ultimately causing cellular dysfunction and death [60]. Notably, these toxic ROS cannot be effectively scavenged by the intracellular antioxidant enzyme system [61], leading to cellular membrane damage, leakage of intracellular potassium ions, and alterations in cellular morphology [62], all of which contribute to the death of microorganisms.

**Figure 6:**
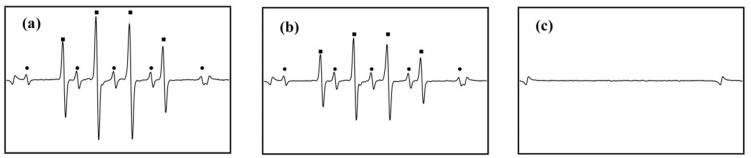
ESR spectra of DMPO after different irradiation (**a**) MW combined with UV; (**b**) UV; (**c**) MW [26].

In the study detailed in Figure 6, Shiro and co-workers [26] revealed that the simultaneous application of microwaves and UV radiation can substantially boost the production of ROS, such as ·OH and ·O_2_^−^, detected via the electron spin resonance (ESR) technique. This investigation involved treating a 5,5-Dimethyl-1-pyrroline-N-oxide (DMPO) solution with MW, UV, and a combination of both under controlled conditions. The ESR signal comprises signals from DMPO-OH adducts, indicated by filled squares, and DMPO-H adducts, represented by filled circles. The findings demonstrated a significant increase in ROS generation when the solution was simultaneously irradiated with MW and UV light, compared to the sole application of UV and MW. Additionally, the study provided evidence that MW is able to enhance the emission of UV photons, which leads to increased ROS production facilitated by the photon absorption by iron (III) oxalate. This synergistic effect is particularly critical in the field of sterilization, offering a superior method for the effective eradication of microorganisms.

**Figure 7:**
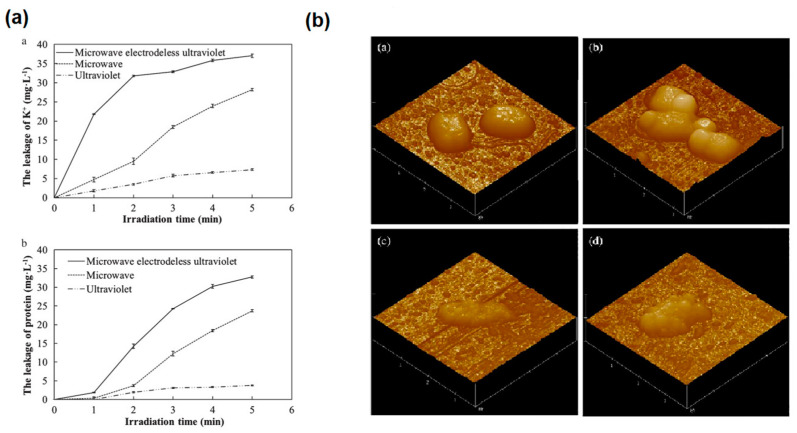
(**a**) Leakage of *E. coli* under different treatments: a. K^+^ leakage, b. protein leakage. (**b**) AFM images of *E. coli*: a. normal *E. coli*, b. UV treatment for 3 min, c. microwave treatment for 1 min, and d. MWUV treatment for 1 min [63].

**Figure 8:**
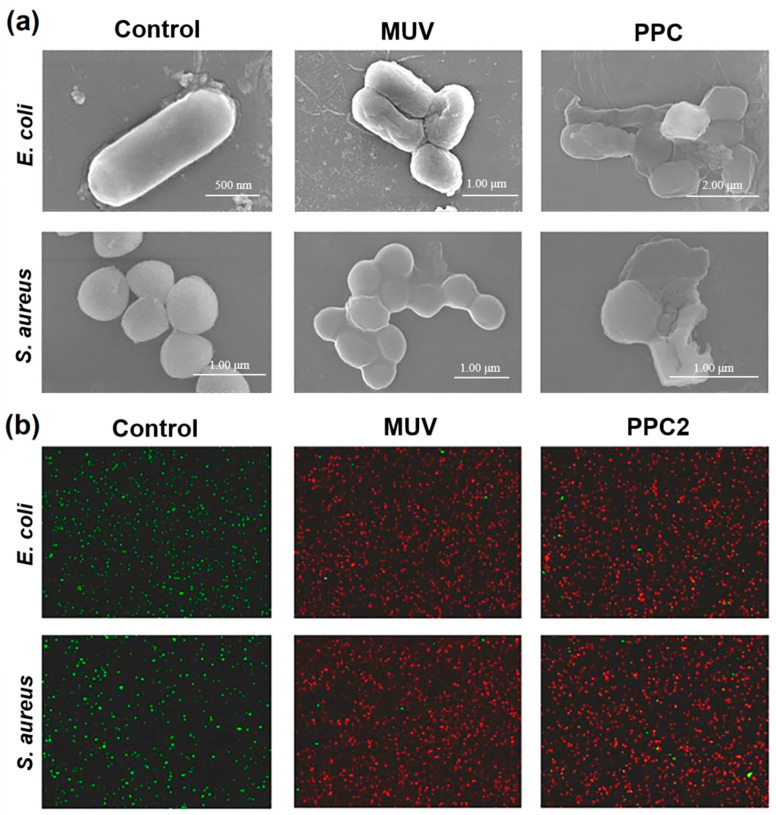
(**a**) Bacterial morphological changes; (**b**) bacterial staining for live and dead [65].

The detrimental effects of MWUV on microbial cellular structures predominantly involve damage to the bacterial cell wall and cell membrane, which leads to the leakage of bacterial endosolutes and the degradation of vital intracellular nutrients, such as proteins. This damage extends significantly to the cellular nucleic acids, DNA, and RNA. The dual protective barriers, comprising the cell wall and membrane, offer varying levels of resilience against different types of irradiation. Specifically, UV radiation impacts the cell membrane minimally, while microwave radiation notably disrupts it, and the synergy of MW-UV irradiation intensifies these effects dramatically. Zhang [63] employed *Escherichia coli* (*E. coli*) as a model organism to track the leakage of intracellular potassium (K^+^) and proteins under varying irradiation scenarios. As depicted in Figure 7, MWUV irradiation leads to the most severe cell membrane damage, far exceeding that caused by MW or UV alone. Atomic force microscopy (AFM) images post three minutes of UV exposure show that the *E. coli* surface became rough, and the edges turned irregular, but the overall bacterial shape was maintained. In contrast, a single minute of MW exposure leads to a profoundly uneven surface and extremely irregular edges, resulting in the organism’s collapse and shrinkage and the leakage of intracellular materials, ultimately leading to a complete loss of the original shape. The effects of one minute of MWUV irradiation on *E. coli* are akin to those observed with microwave treatment but with an even more pronounced loss of structural integrity. This result is primarily due to the MW’s thermal and non-thermal effects, which compromise the integrity of the cell membrane, and this extensive damage is further exacerbated by the production of ROS by UV, which enhances the degradation of the cell membrane [64]. Wang et al. [65] further explored these cytoarchitectural changes through scanning electron microscopy (SEM) and live/dead staining methods, comparing the bactericidal effects of MWUV and combined MWUV with ultrasound (US), named PPC. As depicted in Figure 8a, the cell surface morphologies of *E. coli* and *Staphylococcus aureus* (*S. aureus*) post-treatment with MWUV and PPC both exhibit noticeable irregularities when contrasted with the smooth surfaces of untreated control cells. This visual evidence strongly suggests that treatments involving MWUV can severely compromise the structural integrity of the bacterial cell walls. The images in Figure 8b demonstrate a shift from densely packed green fluorescent spots, typical of healthy control cells, to predominantly red fluorescent spots followed by MWUV and PPC treatments; this shift indicates a significant reduction in bacterial viability. Moreover, the disruption of bacteria structures caused by PPC was notably more severe than that caused by MWUV, and the intensity of red fluorescence increased substantially under PPC treatment, pointing to a more extensive bacterial inactivation. These findings suggest that introducing the turbulence effect of US and integrating it with the bactericidal capability of MWUV can enhance the sterilizing efficiency of MWUV for non-transparent sample sterilization, such as milk, through a synergistic effect.

MWUV inflicts damage on nucleic acids, including DNA and RNA, through mechanisms that are both direct and indirect. Direct damage occurs predominantly when UV light is absorbed by nucleic acids, leading to covalent bonding between adjacent pyrimidine bases in DNA, most notably thymine bases, which often leads to the creation of thymine dimers. These dimers disrupt the natural double helix structure of DNA, complicating the processes of DNA replication and transcription. Additionally, UV light facilitates the production of 6-4 photoproducts between thymine and adenine, further altering the structural integrity of DNA [66]. RNA, which is similarly susceptible to UV light, experiences disruption in its base pairs and the formation of pyrimidine dimers. Furthermore, UV light compromises the tertiary structure of RNA, affecting its proper spatial configuration and thus potentially impairing its roles in transcription and messenger functionality [67]. Indirect damage to nucleic acids is chiefly oxidative, precipitated by the generation of ROS such as superoxide, hydrogen peroxide, and hydroxyl radicals under UV irradiation. This oxidative stress wreaks havoc on both DNA and RNA, facilitating base substitutions, additions, deletions, and other mutagenic alterations. During this oxidative assault, a plethora of oxidized nucleic acid products are produced. Initially, the hydroxyl radical reacts with 2′-deoxyguanosine or guanosine to form a C8-OH-adduct radical. This radical, upon losing an electron and a proton, becomes transformed into 8-oxodG or 8-oxoG [68,69]. These compounds are indicative of oxidative damage to DNA and RNA and are prone to inducing base-pairing errors, leading to mutations and genetic instability. Oxidative stress also undermines the normal functions of RNA, such as ribosomal activity, reducing translation efficiency and causing disruptions in protein synthesis. This includes a decline in protein synthesis rates and the production of misfolded peptides, ultimately resulting in the dysregulation of gene expression [70,71].

**Figure 9:**
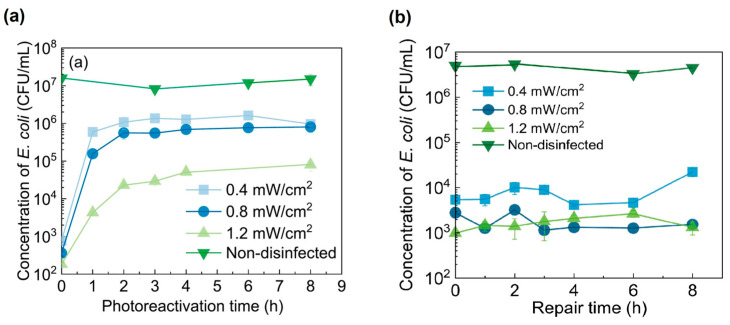
(**a**) *E. coli* changes over photoreactivation time; (**b**) *E. coli* changes over dark treatment time [42].

The phenomenon of bacterial resurgence observed after MWUV sterilization processes may be attributed to the mechanisms that repair photo-induced DNA damage. Various microorganisms have different capabilities to mend DNA damage through both photoreactivation and non-light-dependent dark repair pathways [66]. Photoreactivation involves critical photolytic enzymes that specifically recognize and attach to DNA lesions, such as CPD and 6-4PP, harnessing light energy to effectively reverse the DNA damage. Conversely, dark repair utilizes specific enzymes, including N-glycosylase, which cleaves DNA crosslinks induced by UV exposure, operating independently of light energy [72]. In their experimental findings, Wang [42] has demonstrated, as indicated in Figure 9, that *E. coli* undergoes rapid photoreactivation within the first hour after MWUV disinfection at an intensity of 16 mJ/cm^2^. This rapid recovery then plateaus after about three hours, with no further significant changes in bacterial concentrations, implying that some bacterial damage inflicted by MWUV irradiation is beyond the repair capabilities of photoreactivation. In contrast, under dark conditions, *E. coli* populations in non-disinfected samples exhibited minimal change over an 8 h period, underscoring the minimal impact of dark repair mechanisms compared to photoreactivation. Despite the role of photoreactivation in mitigating the effects of MWUV sterilization, it is the microwave component that predominantly leads to bacterial cell destruction and morphological deformation due to irreversible damage to their cell membranes and walls. This microwave-induced damage significantly neutralizes the potential regenerative effects of UV-induced biological photoreactivation. Therefore, to effectively prevent the occurrence of photoreactivation in practical future applications, it is essential to carefully calibrate the microwave and UV power settings to optimize sterilization efficacy, ensuring maximum bacterial inactivation while minimizing the potential for DNA repair that could lead to bacterial resurgence.

### 3.2. Microbial Decontamination

MWUV has demonstrated significant efficacy in the sterilization of microorganisms present in food products. Typical pathogens found in food include the following.

- *Escherichia coli*: *E. coli*, commonly found in the gastrointestinal systems of both humans and animals, is mostly nonpathogenic [73]. Nonetheless, specific variants such as strains O157:H7 and O104:H4 have been linked to significant outbreaks of foodborne illnesses. Manifestations of infections caused by these strains can range from diarrhea and abdominal discomfort to vomiting and high fevers, which in the most severe cases may advance to renal failure. Furthermore, *E. coli* is notably infectious and can be transmitted through the ingestion of foods or liquids that are contaminated, including raw or insufficiently cooked meats, unpasteurized dairy products, and fresh produce that has not been adequately cleaned. Individuals who contract this bacterium are capable of spreading it to others, thereby facilitating the propagation of the infection.
- *Bacillus subtilis:* Spores of *B. subtilis* are notably resilient to elevated temperatures [74], which enables them to endure the thermal processes commonly utilized in various food preparation methods. These spores maintain their viability even after exposure to the high temperatures involved in such processes, potentially leading to subsequent issues related to food safety due to their survival.
- *Salmonella* spp.: *Salmonella* contamination is predominantly linked to sources such as poultry, eggs, and meat products that have not been cooked thoroughly [75]. During the various stages of production, transportation, and handling, lapses in proper sanitary practices can lead to cross-contamination. This increases the potential for the spread of infectious diseases significantly as pathogens find new pathways to infect hosts.
- *Bacillus cereus: Bacillus cereus* is recognized for causing foodborne illnesses, manifesting either as emetic or diarrheal syndromes. Its widespread presence across various environments, coupled with its capability to form spores and adapt to diverse conditions, allows it to produce potent toxins. These characteristics establish Bacillus cereus as a significant public health risk that demands serious attention [76].

The microbial degradation of bacterial fungi by MWUV is shown in Table 3.

The efficacy of MWUV sterilization in deactivating a wide range of variations in microorganisms, including both Gram-positive and Gram-negative bacteria, has been well-documented. Observational studies, such as those by Cheng et al. [77], report that Gram-negative bacteria, exemplified by *E. coli*, are more susceptible to MWUV inactivation compared to Gram-positive bacteria like *B. subtilis*. Specifically, experimental data indicated that, at a consistent power setting for a duration of 150 s, the inactivation rate of *E. coli* reached 99.02%, while *B. subtilis* was less affected, with a 50.05% reduction. When the duration was increased to 180 s, the inactivation rate for *E. coli* escalated to 99.87%, whereas B. subtilis saw a moderate increase to 54.87%. The introduction of ozone into the MWUV process further enhances its bactericidal effectiveness, particularly boosting the rates of bacterial destruction. At 150 s, the incorporation of ozone increased the inactivation rate of *E. coli* to 99.92% and improved *B. subtilis*’s rate to 80.92%. At 180 s, these rates climbed even higher, with *E. coli* reaching an inactivation rate of 99.99% and *B. subtilis* achieving 86.34%. Further experiments conducted by Wang et al. [42] demonstrated that, under MWUV exposure for merely 16.67 s, *E. coli* experienced a reduction by 4.5 log orders of magnitude, showcasing a high susceptibility. In contrast, *B. subtilis* required 41.67 s to achieve a reduction of 3.4 log orders of magnitude, highlighting its comparatively greater resistance. This disparity is largely due to the structural differences between the bacterial cell walls; Gram-negative bacteria have thinner cell walls with an outer lipid bilayer that allows UV light to penetrate more easily [78,79,80]. Conversely, Gram-positive bacteria possess a substantially thicker peptidoglycan layer, providing more effective protection against direct UV damage and potentially higher capabilities for DNA repair, enabling them to more effectively repair any UV-induced damage to their genetic material. This structural difference results in a varied response to MWUV sterilization, making Gram-negative bacteria generally more vulnerable to such treatments than their Gram-positive counterparts.

**Table 3:** Microbiological degradation of bacterial/fungi by MWUV.

MDEL Types	Microwave Power	UV Power/Intensity	Bacteria/Fungi	Sterilization Samples	Initial Bacterial Concentration	Treated Time	Inactivation Rate/Order of Magnitude Reduction	References
MOTEL(MWUV)	2450 MHZ	N.A	*E. coli*	NaCl solution	2 × 10^4^ (CFU/mL)	10 s	100%	[26]
MOTEL(MWUV)	700 W	3.60 mW/cm^2^	*E. coli*	Water	7.8 × 10^5^ (CFU/L)	150 s	99.02%	[77]
						180 s	99.87%	
			*B. subtilis*	Water	1.2 × 10^5^ (CFU/L)	150 s	50.05%	
						180 s	54.87%	
MOTEL(MWUV)	2.45 GHz	35.3 mJ/cm^2^	*E. coli*	droplet	7.6 × 10^7^ (CFU/L)	3 s	99.9998%	[81]
				film	7.6 × 10^7^ (CFU/L)	3 s	99.9999%	
MOTEL(MWUV)	2.45 GHz	N.A.	*E. coli*	milk	5.408 log (CFU/mL)	20 s	Reduced 5 log (CFU/mL)	[27]
MOTEL(MWUV)	600 W	0.33 mW/cm^2^	*E. coli*	effluent	3400 (CFU/mL)	20 s	100%	[63]
MOTEL(MWUV)	180–900 W	N.A.	*R. dominica*	wheat grains	N.A.	10–60 s	90–100%	[82]
MOTEL(MWUV/O_3_)	700 W	3.60 mW/cm^2^	*E. coli*	Water	7.8 × 10^5^ (CFU/L)	150 s	99.92	[77]
						180 s	99.99%	
			*B. subtilis*	Water	1.2 × 10^5^ (CFU/L)	150 s	80.92%	
						180 s	86.34%	
MOTEL(MWUV/O_3_)	2.45 GHz	22.8 mJ/cm^2^	*E. coli*	droplet	7.6 × 10^7^ (CFU/L)	3 s	99.9998%	[81]
				film	7.6 × 10^7^ (CFU/L)	3 s	99.96%	
RCEL(MWUV)	2.45 GHz	8.97 mJ/cm^2^	*E. coli*	cellulose filter paper	2.36 × 10^7^ (CFU/L)	5 s	99.9989%	[81]
		17.9 mJ/cm^2^	*E. coli*	cellulose filter paper	2.36 × 10^7^ (CFU/L)	5 s	99.9999%	
RCEL(MWUV)	N.A.	1.6 mW/cm^2^ 20 mJ/cm^2^	*E. coli*	Bacterial suspension	Approximately 10^6^/10^7^ (CFU/mL)	16.67 s	Reduced 4.5 log (CFU/mL)	[42]
		1.2 mW/cm^2^/50 mJ/cm^2^	*B. subtilis*	Bacterial suspension	Approximately 10^6^/10^7^ (CFU/mL)	41.67 s	Reduces 3.4 log (CFU/mL)	
RCEL(MWUV)	2.45 GHz	20 J/m^2^	*E. coli ATCC 25922*	Nutrient Agar plates	Approximately 10^5^/10^7^ (CFU/mL)	2 s	Reduces 6 log (CFU/mL)	[83]
			*Pseudomonas aeruginosa NCTC 10662*	Nutrient Agar plates	Approximately 10^5^/10^7^ (CFU/mL)	2 s	Reduces 6 log (CFU/mL)	
		600 J/m^2^	*Bacillus cereus spores*	Nutrient Agar plates	Approximately 10^5^/10^7^ (CFU/mL)	10 s	Reduces 2 log (CFU/mL)	
			*Staphylococcus aureus NCNTC 6571*	Nutrient Agar plates	Approximately 10^5^/10^6^/10^7^ (CFU/mL)	2 s	Reduces 6 log (CFU/mL)	
RCEL(MWUV/US)	N.A.	20 mW/cm^2^	*coliforms*	Raw milk	Approximately 6.1 × 10^6^ (CFU/mL)	30 min	Reduced 4.8 log	[65]
			*yeast and mold*	Raw milk	Approximately 6.8 × 10^6^ (CFU/mL)	30 min	100%	

“N.A.” refers to the fact that the data were not provided in the original literature.

## 4. Applications of MWUV in the Food Field

### 4.1. Sterilization and Storage

1. Dairy products

As health concerns continue to rise, dairy products are increasingly recognized as a premium source of protein and calcium [84]. Dairy products are also abundant in various essential nutrients, including phosphorus and vitamins. Protein is crucial for issues development, while calcium and phosphorus play significant roles in the healthy development of bones and teeth [85,86]. Furthermore, the vitamins present in dairy contribute substantially to functions such as immune response and vision. However, dairy products are characterized by a high water, fat, and protein content, which create a conducive environment for microbial growth. Typically, raw milk is particularly vulnerable to spoilage over relatively short periods. Microorganisms’ metabolic activity causes the breakdown of sugars and proteins in milk, resulting in acidification and undesirable flavors [87]. As for effectively prolonging dairy products’ shelf life, the main common sterilization methods employed include pasteurization [88], ultrahigh-temperature sterilization (UHT) [89], and Pulsed Electric Field (PEF) technology [90].

Singh et al. [27] utilized MWUV for milk sterilization and revealed that the sterilization system was more effective than microwave sterilization, as shown in Figure 3a. The two treatments exhibited a statistically significant difference (*p* < 0.05) in terms of total colony count and methylene blue reduction test within the treatment duration of 5 to 15 s. MWUV achieved an exponential reduction in microbial counts to less than 1 Log (CFU/mL) after a 20 s treatment. The physicochemical properties of milk, such as pH, color, and total soluble solids (TSS), did not undergo significant alterations during this process. Wang et al. [65] discovered a novel photon-phonon coupling cold sterilization technology (PPC) and investigated its effects on microbial communities in milk, the sensory attributes of dairy products, nutrient composition, and whey proteins. The MWUV intensity was 20 mW/cm^2^, and the ultrasonic output powers were 600 W and 1200 W, corresponding to the energy densities of 1405 and 2810 J/mL, respectively. The flow velocity of milk in the equipment pipelines was 180 L/h. The study also employed simulated low-temperature pasteurization (SLP) at 65 °C temperature for 30 min, along with microwave ultraviolet (MUV), as two comparative technologies for assessment. It demonstrated that PPC technology effectively decreased the microbial counts in raw milk (by approximately 4.8 log CFU/mL) while minimally affecting color, taste, and odor, relying on retaining the main nutrients like lactoferrin and immunoglobulins. PPC exerted a lesser effect on the structural aspects, surface charge, and hydrophobicity of whey proteins than conventional low-temperature pasteurization.

2. Fruits and vegetable products

It is normal for fresh fruits and vegetables to host non-pathogenic, epiphytic microflora. Although this microbial presence does not pose a direct danger to human health, it may lead to a reduction in the product’s lifespan [91]. As a result, various decontamination protocols are routinely implemented for these items. Nevertheless, there are occasions when these products may become contaminated. Such contamination can occur during agricultural production (for instance, the utilization of contaminated irrigation water or raw manure), as well as at any stage of the handling process, ranging from harvest to sale (including processes such as washing, cutting, slicing, and packaging) [91].

To date, MWUV has not been directly applied to the sterilization of fresh fruits and vegetables. However, studies have been conducted on the microbiological assessment of products processed using vegetables and fruits as raw materials. Gómez-Sánchez [92] examined the efficacy of a continuous-flow UV-Microwave system for sterilizing *S. cerevisiae* and *E. coli* in pomegranate juice. The UV–MW system assessed at a flow rate of 400 mL/min demonstrated superior effectiveness in inactivating both yeast and bacteria, the MW was 1000 W energy output, 2450 MHz and UV power was 14 W, achieving approximately six cycles of log reductions following three passes through the system. Meanwhile, it has been demonstrated that UV-C has a pronounced effect on postharvest fungal infections of fruits and vegetables. For instance, it has been reported that UV-C treatment can markedly inhibit the germination and sporulation of Colletotrichum in mango [93]. Moreover, recent studies have demonstrated the efficacy of UV as an inhibitory treatment for postharvest fruit and vegetable senescence. It possesses the potential to prolong the shelf life and maintain the quality of postharvest fruits and vegetables, including slowing chlorophyll degradation in green produce and mitigating chilling damage resulting from cold storage [94]. Therefore, it is anticipated that MWUV, a synergistic antimicrobial effect of MW and UV, will demonstrate superior efficacy in the preservation of fruit and vegetables.

3. Grains

Grains can be affected by various bacterial and fungal contaminants during storage. Particularly under conditions of high humidity and inappropriate temperatures, the proliferation of pathogenic microorganisms can result in food spoilage and toxin production [95]. Common bacterial contaminants found in food include *Bacillus* and *Macrobacterium*, which may contribute to spoilage under suboptimal storage conditions, thereby compromising food quality. Species such as *Aspergillus species* (including *Aspergillus flavus* and *Penicillium*) are among the most prevalent molds associated with food products. These molds not only induce spoilage but also produce mycotoxins that pose significant health risks to both humans and animals, like aflatoxins. Numerous studies have demonstrated that mycotoxins have carcinogenic, mutagenic, and teratogenic effect types on both humans and animals alike. They may also infect edible animals with transmissible diseases, potentially leading to reduced productivity within the livestock sector while facilitating pathogens transmission to humans. Therefore, it is of essence to carry out measures that prevent mold in food.

Srivastava and co-workers [82] explored the use of MWUV combined with vacuum technology (MWUV/VC) for sterilizing stored rice grains; the MW power ranged from 0 W to 1000 W, and the UV power was 300 W. The study showed that this combination effectively eliminated microorganisms. Importantly, the MWUV/VC had a minimal effect on the moisture content, fat levels, ash, protein, hardness, bulk density, true density, and other quality aspects of rice compared to traditional sterilization methods. As a non-chemical treatment option, MWUV can help improve the future storage of rice and other grains without leaving chemical residues or significantly impacting the environment. This method is quite effective in maintaining grain quality throughout the supply chain process.

Table 4 summarizes the applications of MWUV in the food field. At present, it is mainly focused on the sterilization of fluid foods and the surface sterilization of solid foods. The future research direction can be extended to powdered foods, such as milk powder, but it is key to solve the poor penetration of UV light. The above reports have demonstrated the potential of MWUV as a new sterilization technology. However, it still remains at the laboratory scale. In the actual industrial field, it is critical to design different industrial-scale-up equipment with reference to the features of food. For example, for liquid foods with high turbidity, like milk and fruit juice, it is possible to connect several continuous-flow UV-microwave reactor devices in the production line to ensure the uniform distribution of MW and UV light and high throughput. For solid foods such as cheese and grains, it is important to design MWUV treatment equipment or treatment methods that are suitable for solid foods with different shapes and sizes. For instance, it is necessary to consider the development of special conveyor devices or treatment chambers to ensure effective sterilization treatment for each solid food item and all its sides. In our laboratory, research on the sterilization of Mongolian milk tofu by MWUV has been carried out and achieved ideal results. The equipment with flip and transfer functions is being self-developed in our laboratory to meet the high throughput demand in industrial applications.

Nevertheless, currently, there is a lack of unified operation specifications and certification standards for the application of MWUV technology in the food industry. Meanwhile, for foods with complex internal structures (such as multi-layered sandwich foods and foods with porous structures), MWUV technology still has limitations in terms of sterilization uniformity and effectiveness. In the future, it will be necessary to improve the technology or treatment methods to overcome these problems.

4. Food contact materials

Food contact materials (FCMs) involve all types of products that come into contact with food during its production, processing, packaging, storage, transportation, or consumption. These materials must adhere to rigorous safety and hygiene standards to guarantee their safety for human health. The surfaces of FCMs are highly susceptible to microbial contamination, which may act as a source of infection or facilitate the spread of disease dissemination. Microorganisms on these surfaces can exist either as single-cell entities or well-developed biofilms. Beyond contributing to the transmission of diseases, biofilms formed on food contact surfaces can also shorten the shelf-life of products and modify their appearance [81].

However, the number of studies investigating its potential for sterilizing the food contact material surface is limited. Sheng et al. [96] developed a cold plasma combined with a +222 nm ultraviolet (like CP+UV) sterilization device. After 60 s of this treatment, *S. aureus* counts on the glass sheets, polypropylene films, corrugated paper, and kraft paper were reduced by 4.5, 4.1, 1.5, and 2.4 log CFU/cm^2^, respectively. Livingston et al. [97] proved that exposure to UV-A light led to slight methicillin-resistant *Staphylococcus aureus* (MRSA), Candia auris (Centers for Disease Control and Prevention strain 0381), the nonenveloped virus bacteriophage MS-2 (ATCC 15597-B1), and the enveloped virus bacteriophage Phi X174 (ATCC 13706-B1) reductions, which were on steel disk carriers after several hours of treatment. Barkhudarov et al. [81] evaluated the effectiveness of MWUV kill against *E. coli* presented as films, droplets, or dried bacteria on cellulose filter membranes for over 2 h. Bacteria on cellulose filter membranes decreased by 5 log CFU/cm^2^ in 5 s at an 8.97 mJ/cm^2^ UV dose. Meanwhile, *E. coli* in droplet and film forms were reduced by 5–6 log CFU/cm^2^ in just 3 s at a 35.3 mJ/cm^2^ UV dose. In the future, it will be valuable to develop the application of MWUV for food contact materials.

### 4.2. Food Detection

The analysis of food elements is a crucial step in guaranteeing food safety and quality. The accurate determination of essential and toxic elements relies on the digestion process employed for the analysis of the examined sample [98,99]. Traditional food digestion methods comprise wet digestion [100], dry aching [101], etc. Wet digestion dissolves food samples by heating it with nitric acid, sulfuric acid, or hydrogen peroxide. Dry aching incinerates the sample at an elevated temperature to produce ash, which is subsequently dissolved in acid for elemental analysis.

Microwave-assisted digestion is an advanced approach that leverages microwave heating technology. By mixing the sample with acid (like nitric acid, hydrogen peroxide, etc.) and placing it in a closed vessel, microwave radiation promptly creates an elevated temperature and high-pressure environment, decomposing complex organic matter and releasing inorganic elements. Compared to traditional methods, it is faster and more efficient, uses less acid, and mitigates pollution risks. It is frequently employed in the analysis of heavy metals in food samples. Ibrahim et al. [102] developed a closed-vessel microwave digestion protocol to efficiently and accurately determine macro, micro, and toxic elements in wheat, which include Mg, Mn, Se, Cd, As, etc., followed by analysis of inductively coupled plasma mass spectrometry (ICP-MS). Multiple samples were analyzed with analytical models, principal component analysis (PCA), and hierarchical cluster analysis (HCA) to understand their similarities. Henn et al. [103] used microwave-assisted solid sampling analysis coupled with flame furnace atomic absorption spectrometry (MW-SS-FF-AAS) for detecting the presence of Cd and Pb in polymer samples intended for food contact. The results indicated that this digestion method had no significant difference from certified values and correlated well with traditional digestion methods, as well as saving concentrated acid utilization to reduce waste.

UV excitation engenders a distinctive chemical milieu and significantly affects numerous reactions. UV radiation catalyzes the digestion of added oxidizing agents, yielding reactive radicals and various organic substances, including alkanes, haloalkanes, aliphatic alcohols, carboxylic acids, alkenes, aromatics, phenols, pesticides, herbicides, dyes, and surfactants, among others, that are susceptible to degradation via UV oxidation [104].

The combination radiation of UV and MW constitutes an alternative method for degrading organic species through oxidation, facilitated by chemical reactions involving molecules that have been electronically excited upon light absorption [104], which was originally reported by Florian and co-workers [105]; a MDEL was directly placed inside the digestion vessel to initiate UV radiation emission under a microwave field. The employment of UV radiation during sample digestion confers substantial advantages. In the presence of oxygen or hydrogen peroxide, high amounts of ROS are generated, which accelerate the decomposition of organic substances. All the applications developed for sample digestion typically incorporate additional reagents, such as HNO_3_ and/or H_2_O_2_. Owing to the higher temperatures in closed pressurized digestion vessels as opposed to open vessels, samples are digested more efficiently. MWUV digestion proves highly efficacy for the digesting homogenous phases, namely liquid samples. For solid samples, however, a diminished efficiency can be accounted for by shielding the effects of solids on the UV radiation. At elevated temperatures around 250 °C in pressurized digestion vessels, solid biological particles dissolve readily, allowing them to be attacked more efficiently by radicals.

MWUV digestion proves to be an effective technique for determining essential and toxic elements in foods, playing a pivotal role in quality control. A comparison between actual and theoretical element detection using this method is presented in Table 5, while Table 6 details the detection of elements in food samples. Nascimento et al. [49] proposed MWUV digestion to analyze both essential and toxic elements. Through MWUV radiation along with HNO_3_, H_2_O_2_, and O_2_, they digested skim milk powder and starch to identify metal elements like Cg, Ca, and Mn. The limits of detection were low, with accuracy deemed satisfactory (>90 %), indicating that the proposed MWUV digestion provides an environmentally friendly method of quality control in milk powder. Cerveira et al. [106] made use of the MWUV method to determine the levels of As, Cd, Hg, and Pb elements in rice and wheat via SF-ICP-MS and CVG-AAS with HNO_3_ as a digestion solution. Hartwig and co-workers [107] assessed the MWUV system for margarine, followed by the determination of Ni, Pd, and Pt elements using ICP-MS. The same method also was used to analyze the As, Cd, Ni, and Pb elements in chocolate, and the MWUV allowed the use of diluted HNO_3_ at 4 mol/L to digest, weighing up to 600 mg [108]. The MWUV method allows for a decrease in the usage of concentrated acids or other oxidizing reagents during digestion, leading to a reduction in chemical waste. Additionally, it helps significantly minimize the interference during the determination by yielding lower blank values.

Rastogi and co-workers [109] presented a novel technique known as MWUV-IC for measuring total nitrogen in cereals. In this process, the nitrogen within grains was converted into NH_4_^+^, NO_3_^−^, and NO_2_^−^, using HCl, H_3_BO_3_, and H_2_O_2_ under MWUV digestion; these substances were subsequently quantified through ion chromatography(IC). The total nitrogen results obtained via the MW-UV-IC method closely matched those from the Kjeldhal method while adhering to green chemistry principles. Mesko et al. [110] employed MWUV in conjunction with HNO_3_ to digest seaweed samples and detected As, Cd, and Pb elements by using ICP-MS. The MWUV method was also compared to microwave-assisted wet digestion (MW-AD), which yielded lower limits of detection (LODs). Concurrently, the MWUV method circumvents the utilization of concentrated acids and markedly diminishes the potential for interference with the measurement phase and the generation of laboratory waste, which aligns with the tenets of green chemistry.

## 5. Conclusions

In summary, as food safety and quality requirements continue to increase, the advancement of innovative food processing technologies has garnered significant attention. MWUV sterilization technology as a non-contact approach has particularly attracted interest in food processing due to the synergistic sterilizing effect of MW and UV irradiation. This study provides a systematic review of MWUV equipment types based on the luminescence mechanisms and the sterilization mechanisms of MWUV. MWUV primarily plays the role of sterilization by producing ROS, inducing oxidative stress damage to bacterial cell membranes, and causing leakage of intracellular solutes. Concurrently, MWUV damages nucleic acids, resulting in defective gene expression. Currently, research on the application of MWUV in food science and industry is rarely reported. Thus, there is a promising potential for MWUV utilization in food sterilization, processing, and detection.

1. Adjust the parameters of the MWUV sterilization process to accommodate various food processing requirements. UV sterilization can only target the food surface, while MW penetrates into the food inside. By fine-tuning the intensity parameters of MW and UV treatments, the sterilization potential of each can be fully utilized, achieving more effective food sterilization with minimal effects on the food’s natural quality;
2. Develop efficient industrial food sterilization equipment. MWUV sterilization equipment mostly remains in the laboratory phase. Thus, designing appropriate pipeline systems is essential to promote MWUV applications in the industrialization of food sterilization;
3. Continue to optimize the design of MDEL. When MW energy is converted into UV light, some energy is transformed into heat. Thus, an efficient conversion structure is needed to improve MW energy conversion to UV light. For example, optimizing waveguide design, ensuring complete cavity sealing, and adjusting MW emission frequency can all enhance conversion efficiency;
4. Explore the synergy of MWUV with diverse sterilization techniques. Relying on a single technology can lead to suboptimal bactericidal effects and bacterial resistance. Integrating MWUV with various sterilization methods can maximize the advantages of individual technologies and create a satisfying synergistic sterilization effect.

## Figures and Tables

**Table 1:** The four types of MWUV equipment.

MWUV Types	Characteristics	Advantages	Disadvantages
Microwave oven reactor	The earliest MWUV equipment; placed an MW and UV lamp in the same microwave oven; the MW and UV usually co-worked.	Simple operation, low technical difficulty, convenient construction of equipment platform, and suitability for multiple chemical reaction scenarios.	Not suitable for large industrial applications because of the limit by microwave oven’s size; low energy utilization rate; difficult to precisely control the parameters of MW and UV.
Continuous-flow UV-Microwave reactor	The circulating flow reactor structure allows the liquid reactants to flow continuously through the MW heating and UV radiation zones.	Avoiding overheating and enhanced the efficiency and stability of the reactor; optimize reaction conditions by controlling the flow rate.	The reaction efficiency is reduced and time-consuming under the condition of a lower flow rate; difficult to clean up the continuous-flow reactor, during the switching process of different reactants.
Coaxially driven MWUV reactor	Incorporating a coaxial slot structure; two types of main structures: cylindrical slot waveguide structure and rectangular slot waveguide structure.	The energy coupling between MW and UV light more efficient; achieving miniaturization of device size without compromising on power.	Complex structures lead to high costs and require hard maintenance; demand a relatively high working environment.
Complete ultraviolet reactor	Non-thermal sterilization equipment; MW was only used to excite the UV generation and shield via unique materials; UV can penetrate the unique materials and be utilized.	Compared with traditional UV, the intensity and stability of UV light have both been enhanced.	Limited penetrating ability, making it challenging for UV to reach the interiors of opaque or dark-colored objects.

**Table 2:** The emission wavelengths of MDEL.

Filling Gasses	Wavelength/nm	References
Cd, Ar	228 nm, 326 nm	[49]
Hg, Ar	185 nm, 253.7 nm, 313 nm, 365 nm	[50]
I_2_, Kr	178.3 nm, 180.1 nm, 183 nm, 184.4 nm, 187.6 nm, 206.2 nm, 342 nm	[50]
K, Cl_2_	222 nm, 258 nm	[51]
F, He	157 nm, 166 nm	[52]
N_2_	295 nm, 313 nm	[53]

**Table 4:** Applications of MWUV in the food field.

Food State	Food	Treatment Method	Parameters	Test Indicators	Effects	References
Fluid	Raw milk	MWUV	2450 MHz, 1200 W	Total plate count (TPC), *coliforms* count, pH, color, total soluble solids (TSS)	MWUV decreased the microbial count exponentially and showed <1 log (CFU/mL) after 10 s and no significant difference was observed in the physicochemical properties of milk.	[27]
	Raw milk	MWUV; PPC (MWUVcombined US)	MWUV: 20 mW/cm^2^; US output power: 600 W or 1200 W, corresponding to the energy densities of 1405 and 2810 J/mL	*S. aureus*, *E. coli*, yeast and mold, color, milk taste attributes, smell analysis, fat, protein, lactose, solids-not-fat (SNF), total solids (TS), native immunoglobulins (IgG, IgA, and IgM), lactoferrin (LTF), structure and properties of whey proteins.	MWUV and PPC both have improved the sterilization efficiency compared with low-temperature pasteurization and enhanced the quality of raw milk; PPC showed a bacterial reduction in raw milk by as high as 4.8 log, while indicating slight impacts on the color, taste and smell of raw milk, the main nutrients, as well as lactoferrin and immunoglobulin.	[65]
	Pomegranate juice	UVC-MW	MW: 2450 MHz 1000 W; UVC: 14 W	*S. cerevisiae* and *E. coli.*	Showing synergistic effect between MW and UV; Yielding 6.1 and 5.5 log-cycle reductions for the yeast and bacteria after UVC–MW treatment with a flow rate of 400 mL/min, respectively.	[92]
Solid	Stored grain (rice)	MWUV or MWUV/VC	MW: 0–1000 W; UV: 300 W	*R. dominica*, moisture content, fat levels, ash, protein, hardness, bulk density and true density.	MWUV/VC treatment was much better than the other two approaches (MW and MWUV treatment) in terms of *R. dominica* mortality rate (%) and leads to minimal changes in the quality attributes of the grain.	[82]

**Table 5:** Comparison of MWUV digestion actual and theoretical detection of elements.

Digested Samples	Auxiliary Reagents	Detection Methods	Detection Elements	Certificated Value	Determined Value	References
NIST 8414 (Bovine muscle)	HNO_3_, H_2_O_2_ and O_2_	ICP-OES	Ca	145 ± 20 (μg/g)	127 ± 14 (μg/g)	[49]
			Zn	142 ± 14 (μg/g)	136 ± 2 (μg/g)	
			Cu	2.84 ± 0.45 (μg/g)	2.79 ± 0.10 (μg/g)	
			Fe	71.2 ± 9.2 (μg/g)	66.7 ± 5.9 (μg/g)	
			K	15170 ± 370 (μg/g)	15030 ± 261 (μg/g)	
			Mo	0.08 ± 0.06 (μg/g)	<0.97 (μg/g)	
			Mg	960 ± 95 (μg/g)	922 ± 46 (μg/g)	
			Mn	0.37 ± 0.09 (μg/g)	0.40 ± 0.02 (μg/g)	
			Na	2100 ± 80 (μg/g)	2054 ± 101 (μg/g)	
		ICP-MS	Pb	0.38 ± 0.24 (μg/g)	0.35 ± 0.02 (μg/g)	
			Cd	0.013 ± 0.011 (μg/g)	0.011 ± 0.002 (μg/g)	
BCR 151 (Skim milk powder)	HNO_3_, H_2_O_2_ and O_2_	ICP-OES	Ca	N.A.	13500 ± 250 (μg/g)	
			Zn	N.A.	52.1 ± 0.1 (μg/g)	
			Cu	5.23 ± 0.08 (μg/g)	5.32 ± 0.08 (μg/g)	
			Fe	51.1 ± 1.3 (μg/g)	46.9 ± 4.4 (μg/g)	
			K	N.A.	11130 ± 240 (μg/g)	
			Mo	N.A.	0.273 ± 0.019 (μg/g)	
			Mg	N.A.	1370 ± 28 (μg/g)	
			Mn	N.A.	0.293 ± 0.037 (μg/g)	
			Na	N.A.	3587 ± 37 (μg/g)	
		ICP-MS	Pb	2.002 ± 0.026 (μg/g)	1.87 ± 0.34 (μg/g)	
			Cd	0.101 ± 0.008 (μg/g)	0.091 ± 0.005 (μg/g)	
NIST 1547 (peach leaves)	HNO_3_	SF-ICP-MS	Cd	0.026 ± 0.002 (mg/kg)	0.0248 ± 0.0015 (mg/kg)	[106]
			Pb	0.870 ± 0.002 (mg/kg)	0.8317 ± 0.0116 (mg/kg)	
		CVG-AAS	As	0.060 ± 0.018 (mg/kg)	0.0562 ± 0.0015 (mg/kg)	
			Hg	0.032 ± 0.004 (mg/kg)	0.0315 ± 0.0009 (mg/kg)	
NIST 1573 (tomato leaves)	HNO_3_	SF-ICP-MS	Cd	3 (mg/kg)	2.862 ± 0.094 (mg/kg)	
			Pb	6.3 ± 0.3 (mg/kg)	5.922 ± 0.471 (mg/kg)	
		CVG-AAS	As	0.270 ± 0.05 (mg/kg)	0.2741 ± 0.0090 (mg/kg)	
			Hg	0.1 (mg/kg)	0.1022 ± 0.0062 (mg/kg)	
Margarine with salt (MS-1)	HNO_3_	ICP-MS	Ni	Recovery 97 ± 4%	Recovery 98 ± 2%	[107]
Margarine with salt (MU-1)				Recovery 96 ± 3%	Recovery 98 ± 3%	
Flour solution	H_2_O_2_, HCl, Boric acid	IC	NH_4_^+^	12.5 (mg/L)	12.7 (mg/L)	[109]
			NO_3_^−^	2.5 (mg/L)	2.6 (mg/L)	
			NO_2_^−^	2.5 (mg/L)	2.4 (mg/L)	
CRM 151 (Skimmed Milk)	H_2_O_2_, HNO_3_	ICP-OES	Cd	101.0 ± 8.0 (μg/kg)	110.0 ± 12 (μg/kg)	[105]
			Pb	2.002 ± 0.026 (μg/kg)	1.921 ± 0.045 (μg/kg)	
			Cu	5.23 ± 0.08 (μg/kg)	5.86 ± 0.16 (μg/kg)	
			Fe	50.1 ± 1.3 (μg/kg)	49.8 ± 0.9 (μg/kg)	

“N.A.” refers to the fact that the data were not provided in the original literature. “IC” refers to the ion chromatography. “ICP-OES” refers to the inductively coupled plasma optical emission spectrometer. “ICP-MS” refers to the inductively coupled plasma mass spectrometry. “SF-ICP-MS” refers to the sector-field inductively coupled plasma mass spectrometry. “SF-ICP-MS” refers to the sector-field inductively coupled plasma mass spectrometry. “CVG-AAS” refers to the chemical vapor generation coupled to atomic absorption spectrometry.

**Table 6:** Detection of elements in food by MWUV digestion.

Digested Samples	Auxiliary Reagents	Detection Methods	Detection Elements	References
Starch	HNO_3_, H_2_O_2_ and O_2_	ICP-OES	Ca, Cu, Fe, K, Mg, Mn, Mo, Na, Pb, Zn	[49]
		ICP-MS	Cd, Pb	
White rice, brown rice, parboiled brown rice, parboiled white rice, organic white rice, wheat flour, organic brown rice, wheat grain, brown wheat flour	HNO_3_	CVG-AAS	As, Hg	[106]
White rice, brown rice, parboiled white rice, parboiled brown rice, wheat grain, organic brown rice, brown wheat flour, wheat flour, organic white rice	HNO_3_	SF-ICP-MS	Cd, Pb	[106]
White chocolate, Milk chocolate	HNO_3_	ICP-MS	As, Cd, Ni	[108]
Rice, finger millet, jowar, and pearl millet	H_2_O_2_, HCl, Boric acid	IC	N	[109]
Seaweed	HNO_3_	ICP-MS	As, Cd, Pb	[110]
CRM 151 (Skimmed Milk)	HNO_3_	ICP−OES	Cd, Cu, Pb, Fe	[105]

## Data Availability

The original contributions shown in this study are included in the article, further inquiries can be directed to the corresponding author.
